# Factors associated with prevalent *Mycobacterium tuberculosis* infection and disease among adolescents and adults exposed to rifampin-resistant tuberculosis in the household

**DOI:** 10.1371/journal.pone.0283290

**Published:** 2023-03-17

**Authors:** Soyeon Kim, Anneke C. Hesseling, Xingye Wu, Michael D. Hughes, N. Sarita Shah, Sanjay Gaikwad, Nishi Kumarasamy, Erika Mitchell, Mey Leon, Pedro Gonzales, Sharlaa Badal-Faesen, Madeleine Lourens, Sandy Nerette, Justin Shenje, Petra de Koker, Supalert Nedsuwan, Lerato Mohapi, Unoda A. Chakalisa, Rosie Mngqbisa, Rodrigo Otávio da Silva Escada, Samuel Ouma, Barbara Heckman, Linda Naini, Amita Gupta, Susan Swindells, Gavin Churchyard, on behalf of the ACTG A5300/IMPAACT 2003 PHOENIx Feasibility Study Team

**Affiliations:** 1 Department of Biostatistics, Frontier Science Foundation, Brookline, Massachusetts, United States of America; 2 Faculty of Medicine and Health Sciences, Department of Paediatrics and Child Health, Desmond Tutu TB Centre, Stellenbosch University, Cape Town, South Africa; 3 Center for Biostatistics in AIDS Research, Harvard T.H. Chan School of Public Health, Boston, Massachusetts, United States of America; 4 Hubert Department of Global Health and Department of Epidemiology, Emory University Rollins School of Public Health, Atlanta, Georgia, United States of America; 5 Byramjee Jeejeebhoy Government Medical College CRS and Sassoon General Hospital, BJMC Clinical Research Site, Pune, Maharashtra, India; 6 Chennai Antiviral Research and Treatment (CART), Infectious Disease Medical Center, Voluntary Health Services, Chennai, India; 7 Department of Medicine and University of Cape Town Lung Institute, Division of Pulmonology, University of Cape Town, Cape Town, South Africa; 8 Barranco CRS, Asociación Civil Impacta Salud y Educación, Lima, Peru; 9 San Miguel CRS, Asociación Civil Impacta Salud y Educación, Lima, Peru; 10 University of the Witwatersrand CRS, University of the Witwatersrand, Johannesburg, South Africa; 11 TASK Applied Science CRS, Brooklyn Chest Hospital, Bellville, South Africa; 12 Institute of Infectious Diseases and Reproductive Health, Les Centres GHESKIO, Port-au-Prince, Haiti; 13 South African Tuberculosis Vaccine Initiative, University of Cape Town, Cape Town, South Africa; 14 PHPT-Chiangrai Prachanukroh Hospital, Chiang Rai, Thailand; 15 Soweto CRS, Perinatal HIV Research Unit, University of the Witwatersrand, Johannesburg, South Africa; 16 Gaborone CRS, Princess Marina Hospital, Gaborone, Botswana; 17 Durban Adult HIV CRS, Enhancing Care Foundation, Durban University of Technology, Durban, South Africa; 18 Instituto Nacional de Infectologia Evandro Chagas, Fiocruz, Brazil; 19 Kenya Medical Research Institute, Kisumu, Kenya; 20 Frontier Science Foundation, Amherst, New York, United States of America; 21 Department of Clinical Research and Bioscience, Social & Scientific Systems, Silver Spring, Maryland, United States of America; 22 Department of Medicine, Johns Hopkins University, Baltimore, Maryland, United States of America; 23 Department of Internal Medicine, University of Nebraska Medical Center, Omaha, Nebraska, United States of America; 24 Aurum Institute, Parktown, South Africa, School of Public Health, University of Witwatersrand, Johannesburg, South Africa, Vanderbilt University, Nashville, Tennessee, United States of America; Rutgers Biomedical and Health Sciences, UNITED STATES

## Abstract

**Background:**

Understanding factors associated with prevalent *Mycobacterium tuberculosis* infection and prevalent TB disease in household contacts of patients with drug-resistant tuberculosis (TB) may be useful for TB program staff conducting contact investigations.

**Methods:**

Using data from a cross-sectional study that enrolled index participants with rifampin-resistant pulmonary TB and their household contacts (HHCs), we evaluated HHCs age ≥15 years for factors associated with two outcomes: *Mycobacterium tuberculosis* infection and TB disease. Among HHCs who were not already diagnosed with current active TB disease by the TB program, *Mycobacterium tuberculosis* infection was determined by interferon-gamma release assay (IGRA). TB disease was adjudicated centrally. We fitted logistic regression models using generalized estimating equations.

**Results:**

Seven hundred twelve HHCs age ≥15 years enrolled from 279 households in eight high-TB burden countries were a median age of 34 years, 63% female, 22% current smokers and 8% previous smokers, 8% HIV-positive, and 11% previously treated for TB. Of 686 with determinate IGRA results, 471 tested IGRA positive (prevalence 68.8% (95% Confidence Interval: 64.6%, 72.8%)). Multivariable modeling showed IGRA positivity was more common in HHCs aged 25–49 years; reporting prior TB treatment; reporting incarceration, substance use, and/or a period of daily alcohol use in the past 12 months; sharing a sleeping room or more evenings spent with the index participant; living with smokers; or living in a home of materials typical of low socioeconomic status.

Forty-six (6.5% (95% Confidence Interval: 4.6%, 9.0%)) HHCs age ≥15 years had prevalent TB disease. Multivariable modeling showed higher prevalence of TB disease among HHCs aged ≥50 years; reporting current or previous smoking; reporting a period of daily alcohol use in the past 12 months; and reporting prior TB treatment.

**Conclusion:**

We identified overlapping and distinct characteristics associated with *Mycobacterium tuberculosis* infection and TB disease that may be useful for those conducting household TB investigations.

## Introduction

The World Health Organization (WHO) estimated that 450,000 persons had rifampin-resistant (RR) or multi-drug resistant (MDR)-TB (TB resistant to both rifampin and isoniazid) in 2021 [1]. “The continuing spread of MDR-TB is one of the most urgent and difficult challenges facing global TB control [2].” Household contacts (HHCs) of people with bacteriologically-confirmed pulmonary TB are at substantial risk of developing disease and are a group targeted for TB preventive treatment (TPT), including when the index TB patient has MDR-TB [3]. Because of the lack of efficacy data from randomized trials, TPT recommendations call for “individualized risk assessment and clinical justification” [3]. Yet few studies have assessed the risk of *Mycobacterium tuberculosis* (Mtb) infection and TB disease among HHCs of people with drug-resistant TB. We report on factors associated with prevalent Mtb infection and prevalent TB disease in HHCs age ≥15 years during the initial household contact investigation in a multinational study.

## Methods

### Study design and population

We used data from the PHOENIx Feasibility Study, an observational study that enrolled participants from October 2015 through April 2016, to assess the feasibility of the planned approach and inform the design of the PHOENIx trial [ClinicalTrials.gov Identifier: NCT03568383], a randomized trial evaluating delamanid vs isoniazid for preventing TB in HHCs of MDR-TB, which began enrollment in June 2019. Sites from Africa (Botswana, Kenya, South Africa), Asia (India, Thailand), South America (Brazil, Peru), and the Caribbean (Haiti) planning to participate in the interventional trial enrolled participants. Using a cross-sectional design, adult index participants and their HHCs of all ages were enrolled. Index participants were adults (age ≥18 years) with pulmonary MDR-TB confirmed by culture or molecular testing, or with Xpert MTB/RIF *rpoB* mutation detected pending at the time of enrollment confirmation of resistance to isoniazid and rifampin by drug susceptibility testing (DST) or line probe assay. The index participant must have started appropriate TB treatment within the past 6 months. No details on the index participant’s TB treatment regimen composition or duration were collected.

A HHC was defined as any person living or having lived in the same dwelling unit or plot of land with shared housekeeping arrangements as the index participant and who had reported exposure within 6 months prior the index participant starting MDR-TB treatment. Up to five attempts were made to enroll HHCs from a household. HHCs had to be enrolled within 30 days of their index participant. Although HHCs of all ages were eligible to enroll in the study, for this report we restricted our analysis population to HHCs ≥15 years of age, since data on exposures, and the criteria used for diagnosing TB disease differed for those <15 years (results for HHCs <15 years of age have been reported separately [4]).

### Characteristics of index participants, household contacts, and dwellings

The Feasibility Study collected data on demographics, socioeconomic status, and other factors in the literature associated with Mtb infection or TB disease in individuals exposed to TB (mostly studies in presumed drug-susceptible TB) [5–14]. For index participants, data on medical history (including current TB diagnosis) was collected through review of medical records and participant recall. TB diagnostic methods and tests of infection used varied according to country-specific standard of care. For HHCs, data were collected describing exposure to the index participant, TB history, and TB exposure outside the household, smoking status, and other potential risk factors. Incarceration, period of daily alcohol use, and use of substances had a recall period of 12 months. Household-level data on physical features of the home and smoking were captured.

### Outcomes

#### Mtb infection

HHCs who did not have current active TB diagnosed through the local TB program when presenting for study screening were evaluated for Mtb infection. Mtb infection was determined by interferon-gamma release assay (IGRA) results, using locally available QuantiFERON, either QuantiFERON TB Gold or Gold In-Tube (both Qiagen), following manufacturer’s instructions. While tuberculin skin testing (TST) was also required by the study protocol, it was not done at all sites, largely due to a global reagent shortage when the study was conducted. When both were done, the specimen for IGRA testing was obtained before TST placement.

#### TB disease

HHCs without a current TB diagnosis before study entry were screened for signs/symptoms consistent with TB. A chest X-ray was done if the HHC was not pregnant and one had not done as part of standard of care in the past 30 day. HHCs with symptoms or abnormal chest X-ray had two spot sputa collected for TB diagnostics according to local requirements (e.g., AFB smear, Xpert MTB/RIF, culture, speciation, DST).

A central outcome review group, composed of clinical members of the study team who were not part of the day-to-day management of participants, reviewed data from HHCs if any of the following criteria were met: evidence of TB from culture or molecular test; positive AFB sputum smear; abnormal or uncertain/indeterminate chest x-ray; or signs/symptoms consistent with TB. They reviewed diagnostic test results, signs/symptoms, TST, IGRA, past TB diagnoses, comorbidities (including HIV status), age, and sex. TB disease was defined as confirmed or probable TB by using modified ACTG criteria [15]. Specifically, confirmed TB required a positive result for *Mycobacterium tuberculosis* complex (MTBC) from culture (liquid or solid), Xpert MTB/RIF, or HAIN GenoType MTBDR*plus* and either TB compatible clinical symptoms (pulmonary) or systemic illness plus features of organs involved (extrapulmonary). Probable pulmonary TB required compatible clinical symptoms and at least one of the following 1) positive sputum smear, 2) abnormal chest imaging, or 3) granulomata either positive for AFB or that was caseating; and required that the HHC had no concurrent illness that would explain the findings. Unlike ACTG criteria, TST or IGRA results were not used in defining probable TB disease. Probable extrapulmonary TB required 1) systemic illness plus clinical features of organs involved or granulomata positive for AFB or that was caseating on tissue biopsy from the relevant site; and 2) TB therapy initiated or recommended. For HHCs with current active TB diagnosed by the local TB program before study entry, they reviewed available data from the time of the diagnosis and reviewed their current medical history since TB diagnostic testing and tests of infection were not done as part of study evaluations. HHCs were referred for TPT, TB treatment, or other illness (e.g., HIV infection), as needed per local standard of care.

### Sample size and study power

The protocol allowed 300 index participants and all their enumerated HHCs to enroll. It was anticipated that the enrollment of 300 index participants would provide a reasonable level of information about factors such as household composition and risk factors for TB to guide the development of the PHOENIx trial. Since the study was intended to be descriptive, no formal power calculation was done.

### Statistical analysis

Prevalence of TB disease was estimated using all 712 enrolled HHCs ≥15 years of age. Because participants diagnosed by the local TB program with current active TB disease had limited evaluations and data collection, they were excluded from multivariable modeling to assess risk factors for both Mtb infection and TB disease outcomes. Prevalence of Mtb infection was estimated using HHCs with determinate (i.e., positive or negative) IGRA results.

To estimate prevalence and evaluate risk factors, we fit logistic regression models using generalized estimating equations (GEE) with an exchangeable working correlation to account for clustering within households. If the estimated working correlation was negative (which only occurred when data were sparse), we substituted an independent working correlation. To investigate risk factors, we took an exploratory approach. Multivariable models of Mtb infection and of TB disease included age and HIV status. Other candidate covariates had to be significant at the 0.25 level in a univariable model and were considered using a manual forward selection process and included in the final model if p<0.05. We did not include country of enrollment as a covariate in models but, when data permitted, verified that estimates in the final multivariable models were not significantly affected by its addition. Robust standard errors were used to estimate 95% confidence intervals (CIs). We report nominal two-sided score test p-values for each covariate. For categorical variables with ≥2 degrees of freedom, Wald p-values were provided for pairwise comparisons. All statistical analysis was performed using SAS 9.4 (SAS Institute Inc., Cary, NC, USA).

### Human research participants

Ethical approvals were obtained from each enrolling site’s Institutional Review Board or Ethics Committee (see S1 Table) as per local requirements before screening and enrolling participants. Index participants provided written informed consent for their participation. HHCs or their parent/guardian if age <18 years provided written informed consent, and assent according to local guidelines.

## Results

**Fig 1:**
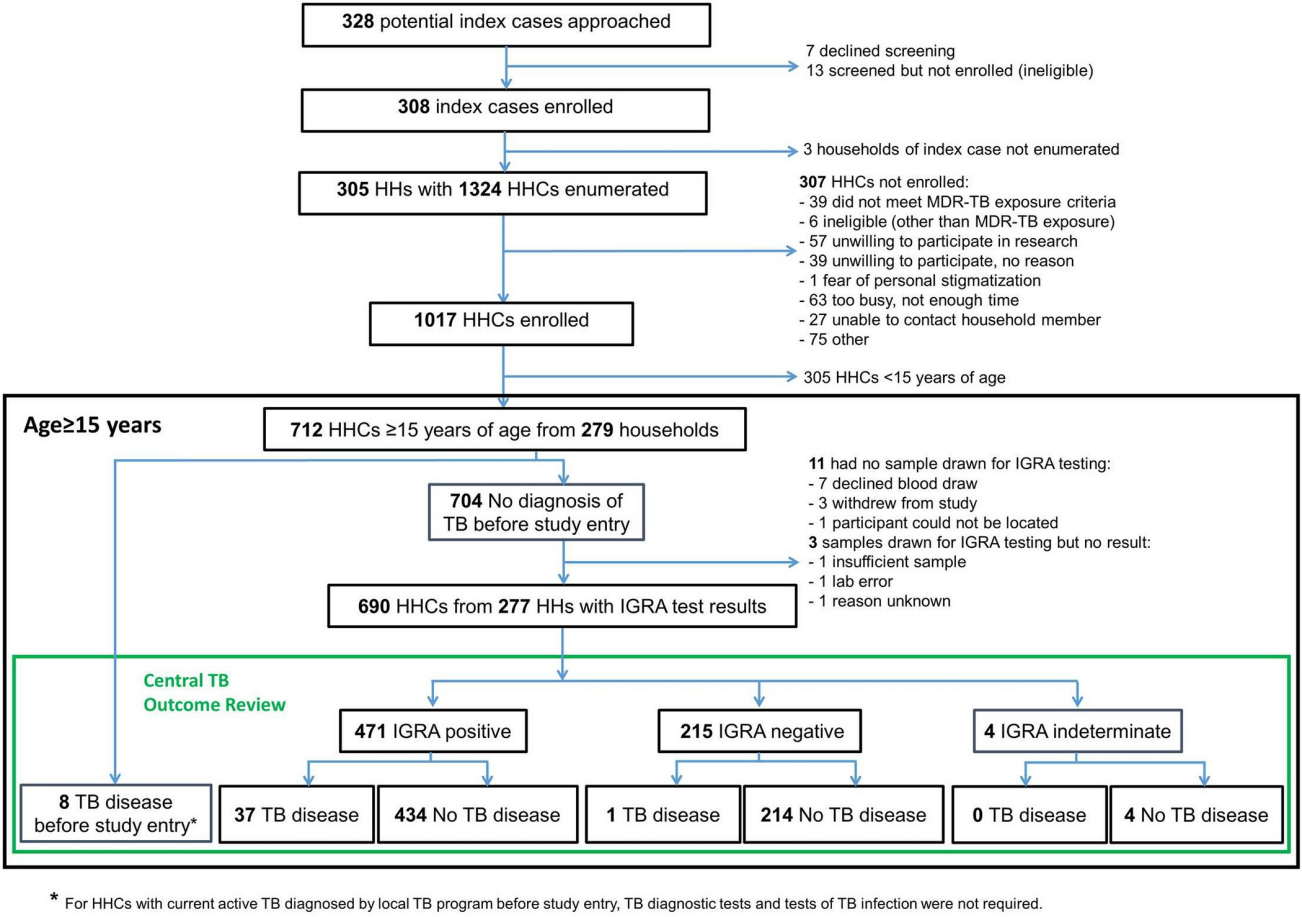
Participant flow diagram. Abbreviations: HH, Household; HHC, household contact; IGRA, interferon-gamma release assay.

Three hundred eight adult index participants and their consenting HHCs were enrolled. Sites in South Africa, followed by India, enrolled the most index participants. Index participants had 1,324 HHCs (including children of all ages), of whom informed consent for study participation was obtained for 1,017. Seven hundred twelve (70%) HHCs were at least 15 years of age and were included in the analysis population (see Fig 1).

All 279 index participants with HHCs ≥15 years of age living in their households had initiated appropriate TB treatment a median of 9 weeks (range: 0 to 27) before enrollment. Time since initiation of TB treatment varied by country (see S2 Table). Index participants were ≥18 years of age as required by the study protocol and a median age of 36 years (range 18, 74). All 279 index participants had *M*. *tuberculosis* resistant to rifampin. Two hundred seven of the 279 (74%) had documented MDR-TB and for 82% (59/72) of the remaining, an isoniazid susceptibility test result was not reported. Fifty-three percent (147/279) of index participants had been treated for TB before the current episode. Seventy-one percent (199/279) lived in houses made of brick, cement, or concrete. Index participants lived in households having a median (interquartile range, IQR) of 5 (3, 6) people. Results of chest x-rays, sputum smear microscopy, and mycobacteriology testing in index participants and characteristics of their households are shown in Table 1.

**Table 1:** Characteristics of household contacts ≥15 years of age and of their index participants and households*.

	Index Participant or Household N = 279		Household Contacts N = 712	
	N	(%)	N	(%)
**Individual Characteristics**				
Country (Number of Sites):				
Botswana (1)	10	(4%)	20	(3%)
Brazil (1)	10	(4%)	17	(2%)
Haiti (1)	14	(5%)	37	(5%)
India (2)	58	(21%)	170	(24%)
Kenya (1)	7	(3%)	12	(2%)
Peru (2)	51	(18%)	130	(18%)
South Africa (7)	120	(43%)	301	(42%)
Thailand (1)	9	(3%)	25	(4%)
Sex:				
Male	163	(58%)	263	(37%)
Female	116	(42%)	449	(63%)
Age (years):				
median (range)	36 (18, 74)		34 (15, 90)	
IQR	(26, 45)		(24, 49)	
15–24	62	(22%)	190	(27%)
25–34	64	(23%)	171	(24%)
35–49	103	(37%)	175	(25%)
≥50	50	(18%)	176	(25%)
Smoking Status:				
Current	62	(22%)	156	(22%)
Previous	62	(22%)	54	(8%)
Never	155	(55%)	502	(71%)
HHC Incarcerated Sometime in Past 12 Mos.^a^				
Yes			20	(3%)
No			684	(96%)
Missing			8	(1%)
HHC Used Alcohol Almost Every Day Sometime in Past 12 Mos.^a^				
Yes			57	(8%)
No			647	(91%)
Missing			8	(1%)
HHC Used Other Substances (Marijuana, Cocaine, etc.) Sometime in Past 12 Mos.^a^				
Yes			58	(8%)
No			651	(91%)
Missing			3	(<1%)
HIV Status:				
HIV-infected	101	(36%)	59	(8%)
HIV-uninfected	150	(54%)	515	(73%)
Unknown	28	(10%)	138	(18%)
History of Previous TB Treatment:				
Yes	147	(53%)	80	(11%)
No/Unknown	132	(47%)	632	(89%)
Duration of Index Participant Treatment at Enrollment (weeks)				
Median (range)	9 (0, 27)		9 (0, 33)	
IQR	(3, 17)		(4, 18)	
TB Preventive Treatment:	NA			
Current			1	(<1%)
Previous			8	(1%)
Never			690	(97%)
Unknown			13	(2%)
Any Signs or Symptoms Consistent with TB for HHC:	NA		
Yes			166	(24%)
No			538	(76%)
Cavitations on Chest X-ray^b^:				
Yes	79	(28%)	7	(1%)
No	102	(37%)	658	(93%)
Unknown	98	(35%)	46	(6%)
Sputum Smear Status (at Diagnosis for Index Participant and Current for HHC)^b^:				
Positive	131	(47%)	13	(2%)
Negative	61	(22%)	170	(24%)
Unknown	87	(31%)	521	(74%)
Confirmation of TB by Phenotypic or Molecular Test:				
Yes	279	(100%)	22	(3%)
No/Not Done	0	(0%)	690	(97%)
Index Participant Drug Susceptibility Profile^b^:				
INH Resistant, RIF Resistant	207	(74%)		
INH Unknown, RIF Resistant	59	(21%)		
INH Susceptible, RIF Resistant	12	(4%)		
INH Indeterminate, RIF Resistant	1	(<1%)		
IGRA Result:	NA		
Positive			471	(67%)
Negative			215	(31%)
Indeterminate/Borderline			4	(1%)
Unknown			14	(2%)
**Household Characteristics**				
Number of Household Members (including Index Participant)				
median (range)	5 (2, 22)			
IQR	(3, 6)			
Dwelling Type:				
Brick, Cement, or Concrete House	199	(71%)		
Tin Shack, Prefab House, or Container	60	(22%)		
Other (Mud, Mud & Stone, Plastic, Sand, Wood, Adobe, Wendy)	18	(6%)		
Unknown	2	(<1%)		
Flooring Material:				
Hardwood; Asphalt or Vinyl Tile; Ceramic Tile; Carpet	118	(42%)		
Planks; Cement; Dirt; Other	159	(57%)		
Unknown	2	(1%)		
Exterior Wall Material:				
Brick, Cinderblock, Stone with Mortar	212	(76%)		
Adobe, Rammed Earth, Sticks/Stones and Mud, Wood, Straw, Tin, Other	65	(23%)		
Unknown	2	(1%)		
Smoking in Household:				
Yes	96	(34%)		
No	178	(64%)		
**Relationship of Index Participant to HHC and Exposure Characteristics**				
Relationship of IP to HHC:				
Spouse			60	(9%)
Son/Daughter			127	(18%)
Mother			55	(8%)
Father			37	(5%)
Other			425	(60%)
Sleeping Proximity of IP to HHC:				
Same Room, Same Bed			84	(12%)
Same Room, Different Bed			156	(22%)
Different Room			464	(66%)
Sleeping Proximity/Evenings per Week with IP:				
Same Room/Any			240	(34%)
Different Room/6-7			327	(46%)
Different Room/3-5			48	(7%)
Different Room/≤2			89	(13%)

Abbreviations and definitions: IP, index participant; HHC, household contact; %, percentage; N, number; IQR, interquartile range (25^th^ and 75^th^ percentiles); range (minimum and maximum); NA, not available; Mos., months; IGRA, interferon-gamma release assay; INH, isoniazid; RIF, rifampin.

Percentages do not always add to 100% due to rounding. Minimum age of index participants was 18 years. HHCs with documented active TB before study entry did not have data collection on incarceration, TB symptoms, or HIV status; they also did not have chest x-ray, specimen collection for IGRA, TB diagnostics, or HIV testing.

^a^ All self reported. Incarceration is defined as having spent a night in jail, prison, youth detention center; alcohol use as period of drinking alcoholic beverages almost every day; and substance use as any use of substances (marijuana, cocaine, etc.). All use a recall period of 12 months.

^b^ For index participants, TB diagnostic data (including chest x-rays, AFB smear results, TB confirmation, and drug susceptibility results) were obtained from routine TB programs performed at the time of TB diagnosis, which occurred before study entry. Test results obtained from routine labs (molecular, phenotypic, or both) are shown. Some programs used Xpert MTB/RIF alone and therefore have only RIF susceptibility results. The majority of index participants did not have susceptibility testing done for any fluoroquinolones or second-line injectable drugs.

Seven hundred twelve HHCs were age ≥15 years and were studied as the target of this secondary analysis. HHCs were enrolled a median of 9 weeks (range: 0, 33) after their index participant started TB treatment. Their median age was 34 years (range 15, 90), 63% (449/712) were assigned female sex at birth (we did not collect information on gender, which might differ from sex assigned at birth), 11% (80/712) of HHCs had been previously treated for TB, 34% (240/712) shared a sleeping room with the index participant, and approximately a third of those sharing a sleeping room also shared a bed (84/240), 29% of HHCs had been exposed (142/712) to someone outside of their household with active TB, of whom half (73/142) were diagnosed before the index participant. Only one HHC was on TPT (0.1%) and an additional eight (1%) had taken TPT for previous TB exposure. See Table 1 for additional details on characteristics of HHCs age ≥15 years and their index participants and households and for selected characteristics by country in S2 and S3 Tables.

### Mtb infection

Of the 704 HHCs age ≥15 years without current active TB diagnosed by the local TB program before study entry and therefore expected to have IGRA testing performed, 14 did not have IGRA test results available and four had indeterminate results. The remaining 686 had determinate IGRA results, and 471 were positive, and the estimated prevalence of Mtb infection based on IGRA was 68.8% (95% CI: 64.6%, 72.8%).

#### Factors associated with Mtb infection

Sleeping proximity and evenings spent with the index participant were correlated; 86% of HHCs who slept in the same room on most nights as the index participant spent seven evenings per week with the index participant, compared to 66% of HHCs who did not sleep in the same room on most nights with the index participant; therefore, these characteristics were combined into a composite HHC-index participant exposure variable. Incarceration, substance use, and alcohol use (each using a 12-month recall period) were also combined into a composite variable but also assessed separately. Table 2 shows results of univariable and multivariable logistic regression models for the prevalent Mtb infection outcome. HHC variables of age, alcohol use almost every day at some time in the past 12 months, the composite of incarceration, substance use, and alcohol use in the past 12 months, smoking history, previous TB treatment, and the household variables of smoking in the household and exterior wall materials had p<0.05 in univariable models.

**Table 2:** Logistic regression modeling for *Mycobacterium tuberculosis* infection (positive IGRA) in household contacts ≥15 years of age*.

Characteristic/Level		All HHCs ≥15 years of age					HHCs ≥15 years of age without previous TB treatment				
		n/N (%) 471/686 (69%)	Univariable		Multivariable		n/N (%) 407/612 (67%)	Univariable		Multivariable	
			Odds Ratio [95% CI]	P value	Odds Ratio [95% CI]	P value		Odds Ratio [95% CI]	P value	Odds Ratio [95% CI]	P value
HHC’s Age	15–24 years	108/184 (59%)	0.7 [0.4,1.0]	**<0.001**	0.7 [0.4, 1.0]	**<0.001**	100/175 (57%)	0.7 [0.5, 1.1]	**<0.001**	0.6 [0.4, 1.0]	**<0.001**
	25–49 years	252/334 (75%)	1.5 [1.0, 2.3]		1.6 [1.0, 2.5]		214/290 (74%)	1.5 [1.0, 2.3]		1.6 [1.0, 2.4]	
	≥50 years	111/168 (66%)	1.0 (ref)		1.0 (ref)		93/147 (63%)	1.0 (ref)		1.0 (ref)	
HHC’s Sex	Male	162/250 (65%)	0.8 [0.5, 1.0]	0.10			137/223 (61%)	0.8 [0.5, 1.0]	0.10		
	Female	309/436 (71%)	1.0 (ref)				270/389 (69%)	1.0 (ref)			
HHC’s HIV Status	HIV-positive	36/56 (64%)	0.8 [0.4, 1.5]	0.43	0.5 [0.2, 0.9]	0.12	25/43 (58%)	0.6 [0.3, 1.3]	0.18	0.4 [0.2, 0.9]	0.11
	Unknown	78/123 (63%)	0.7 [0.5, 1.0]		0.8 [0.5, 1.2]		70/113 (62%)	0.7 [0.5, 1.1]		0.8 [0.5, 1.2]	
	HIV-negative	357/507 (70%)	1.0 (ref)		1.0 (ref)		312/456 (68%)	1.0 (ref)		1.0 (ref)	
HHC’s History of COPD/ Asthma	Yes	24/29 (83%)	1.9 [0.8, 4.8]	0.11			18/23 (78%)	1.6 [0.6, 4.7]	0.34		
	No	442/643 (69%)	1.0 (ref)				385/577(67%)	1.0 (ref)			
HHC Used Alcohol Almost Every Day Sometime in Past 12 Mos.	Yes	46/54 (85%)	2.7 (1.2, 6.1)	**0.004**			32/40 (80%)	2.0 (0.9, 4.5)	0.060		
	No	419/625 (67%)	1.0 (ref)				370/566 (65%)				
HHC’s Smoking History	Current	117/147 (80%)	1.7 (1.2, 2.6)	**0.033**			89/116 (77%)	1.6 (1.0, 2.5)	0.080		
	Previous	36/52 (69%)	1.2 (0.7, 2.0)				25/40 (63%)	0.9 (0.5, 1.7)			
	Never	318/487 (65%)	1.0 (ref)				293/456 (64%)	1.0 (ref)			
HHC’s History of Previous TB Treatment	Yes	64/74 (86%)	2.6 [1.4, 4.7]	**<0.001**	2.1 [1.0, 4.2]	**0.049**	--				
	No/Unknown	407/612 (67%)	1.0 (ref)		1.0 (ref)		407/612 (67%)				
HHC Incarceration, Substance and Alcohol Use Composite in Past 12 Mos.^a^	Incarcerated	19/20 (95%)	6.7 [1.9, 24.4]	**<0.001**	7.3 [1.5, 34.4]	**<0.001**	16/17 (94%)	6.7 [1.6, 27.7]	**<0.001**	7.4 [1.4, 38.5]	**<0.001**
	Never Incarcerated, Used Substances and/or Alcohol	67/80 (84%)	2.3 [1.3, 4.2]		2.6 [1.3, 5.5]		48/60 (80%)	2.0 [1.0, 3.9]		2.6 [1.2, 5.5]	
	Never Incarcerated, No Substances, No Alcohol Use	380/578 (66%)	1.0 (ref)		1.0 (ref)		339/528 (64%)	1.0 (ref)		1.0 (ref)	
Index Participant’s Sputum Smear	AFB-positive	222/314 (71%)	1.2 (0.8, 1.7)	0.38			191/279 (68%)	1.2 (0.8, 1.7)	0.45		
	AFB-negative/ Unknown	314/372 (67%)	1.0 (ref)				216/333 (65%)	1.0 (ref)			
Relationship of IP to HHC	Spouse	37/59 (63%)	0.8 [0.5, 1.5]	0.64			33/55 (60%)	0.8 [0.4, 1.5]	0.54		
	Child	86/123 (70%)	1.1 [0.7, 1.7]				71/103 (69%)	1.2 [0.7, 1.9]			
	Mother	36/54 (67%)	0.9 [0.5, 1.7]				30/48 (62%)	0.9 [0.4, 1.6]			
	Father	28/35 (80%)	1.8 [0.7, 5.0]				26/33 (79%)	1.9 [0.7, 5.2]			
	Other	370/538 (69%)	1.0 (ref)				247/373 (66%)	1.0 (ref)			
Sleeping Proximity of IP to HHC	Same Room	172/233(74%)	1.3 [0.9, 1.8]	0.13			142/201 (71%)	1.2 [0.9, 1.7]	0.29		
	Different Room	299/453(66%)	1.0 (ref)				265/411 (64%)	1.0 (ref)			
Evenings/ Week with IC, Past 6 Mos.	6–7	364/519 (70%)	1.7 [1.1, 2.7]	0.14			313/459 (68%)	1.5 [0.9, 2.5]	0.19		
	3–5	48/71 (68%)	1.5 [0.8, 2.9]				38/61 (62%)	1.2 [0.6, 2.4]			
	≤2	58/95 (61%)	1.0 (ref)				56/92 (61%)	1.0 (ref)			
Sleeping Proximity, Evenings per Week with IC	Same Room/ Any Number	172/233 (74%)	1.9 [1.2, 3.2]	0.10	2.4 [1.4, 4.2]	**0.046**	142/201 (71%)	1.7 [1.0, 2.9]	0.19	2.2 [1.3, 3.9]	0.070
	Different Room/ 6–7	218/320 (68%)	1.7 [1.0, 2.8]		2.1 [1.2, 3.5]		192/287 (67%)	1.6 [1.0, 2.7]		2.1 [1.2, 3.6]	
	Different Room/ 3–5	30/47 (64%)	1.5 [0.7, 3.2]		1.7 [0.7, 4.3]		24/41 (59%)	1.1 [0.5, 2.6]		1.6 [0.6, 4.0]	
	Different Room/≤2	51/86 (59%)	1.0 (ref)		1.0 (ref)		49/83 (59%)	1.0 (ref)		1.0 (ref)	
Smokers in Household^b^	Any	205/267 (77%)	1.8 [1.2, 2.6]	**0.005**	1.6 [1.2, 2.5]	**0.023**	164/222 (74%)	1.6 [1.1, 2.5]	**0.020**	1.6 [1.0, 2.5]	**0.029**
	None	264/410 (64%)	1.0 (ref)		1.0 (ref)		241/381 (63%)	1.0 (ref)		1.0 (ref)	
Home Primary Exterior Wall Material	Brick, Cinderblock, Stone with Mortar	356/534 (67%)	1.0 (ref)	**0.022**	1.0 (ref)	**0.007**	306/476 (64%)	1.0 (ref)	**0.020**	1.0 (ref)	**0.004**
	Adobe, Rammed Earth, Sticks/ Stones & Mud, Wood, Straw, Tin, Other	114/148 (77%)	1.6 [1.1, 2.5]		1.9 [1.2, 2.9]		100/132 (76%)	1.7 [1.1, 2.6]		2.0 [1.3, 3.2]	

Abbreviations: IP, index participant; HHC, household contact; n, number of HHCs with positive IGRA result; N, number of HHCs with determinate IGRA result (positive or negative); %, simple percentage calculated as n/N; CI, confidence interval; ref, reference.

* Models use data from HHC ≥15 years of age with determinate IGRA results; eight HHCs with current active TB documented before study entry did not have specimen collection for IGRA, data collection on incarceration, TB symptoms, or HIV status; they also did not have chest x-ray, TB diagnostics, or HIV testing. Models were fit using an exchangeable working correlation, unless working correlation was negative (which occurred when data were sparse) in which case an independent working correlation was used. Univariable models include the one characteristic only. Each multivariable model includes characteristics with the odds ratios shown. The p values are from score tests.

^a^ All self-reported using a recall period of 12 months. Incarceration is defined as having spent a night in jail, prison, youth detention center; alcohol use as having a period of drinking alcoholic beverages almost every day; and substance use as any use of substances (marijuana, cocaine, etc.).

^b^ Smoking was defined as smoking of tobacco products, ongoing or having quit within the past 6 months.

Multivariable modeling showed that HHCs who reported having previously been treated for TB were more likely to have a positive IGRA result, adjusted odds ratio (aOR) 2.1 (95% CI: 1.0, 4.2) compared to those who reported no or an unknown history of TB treatment. Compared to HHCs who reported not being incarcerated, no substance use, and no period of alcohol use almost every day, those reporting having been incarcerated (with or without having used substances or period of alcohol use almost every day) were most likely to have Mtb infection (aOR = 7.3 (95% CI: 1.5, 34.4)), and those reporting not having been incarcerated but having used substances and/or period of alcohol use almost every day had intermediate Mtb infection prevalence (aOR = 2.6 (95% CI: 1.3, 5.5)). If there were current smokers living in the household (index participant, HHC, or others), the prevalence of Mtb infection in the HHC was higher than if there were no smokers living in the household (aOR = 1.6 (95% CI: 1.2, 2.5)). For the composite HHC-index participant exposure variable, the highest prevalence of Mtb infection was in HHCs who slept in the same room with the index participant (aOR = 2.4 (95% CI: 1.4, 4.2)); in HHCs who did not share a sleeping room with the index participant, the prevalence of Mtb infection was lower and decreased with fewer evenings per week spent together, aOR = 2.1 (95% CI: 1.2, 3.5) for 6–7, aOR = 1.7 (95% CI: 0.7, 4.3) for 3–5, compared to the reference category of ≤2 evenings per week. Compared to exterior walls made of brick, cinderblock, or stone with mortar, the prevalence of Mtb infection was higher in HHCs living in dwellings with exterior walls made of adobe, rammed earth, sticks or stones with mud, wood, straw, tin, or other materials (aOR = 1.9 (95% CI: 1.2, 2.9)). The prevalence of Mtb infection was highest among HHCs 25–49 years of age.

Table 2 also shows models for the prevalent Mtb infection outcome in the subpopulation of HHCs who did not report previous TB treatment. The multivariable model with the same characteristics (except previous TB treatment) had similar adjusted odds ratios as the multivariable model that included HHCs previously treated for TB.

### TB disease

Based on central review, 46 of the 712 HHCs age ≥15 years had prevalent TB disease, including eight with documentation of current active TB diagnosed by the local TB program before study entry (all had microbiologically-confirmed pulmonary TB disease), 14 additional HHCs were adjudicated as having confirmed TB, and 24 as having probable TB. Most (74%, 521/720) HHCs did not have sputum smear microscopy results. The estimated prevalence of TB disease in HHCs age ≥15 years was 6.5% (95% CI: 4.6%, 9.0%). Of those newly diagnosed with active TB disease through study evaluations, all but one of the 14 HHCs with confirmed TB disease and all 24 with probable TB disease had a positive IGRA result. Three of 38 (8%) of HHCs with active TB did not report compatible signs/symptoms of TB. No IGRA testing was done on the eight HHCs with documentation of ongoing active TB through the local TB program at study screening. Among 80 HHCs age ≥15 years who reported previous treatment for TB, the prevalence of TB disease was 30.0% (95% CI: 20.9%, 41.0%). The median time since start of treatment for that previous episode was 7 years and 75% had at least 3 years elapsed since start of treatment (see S2 Table). Among 632 HHCs with no or unknown history of previous TB treatment, prevalence was 3.5% (95% CI: 2.2%, 5.5%). S3 Table shows that eight of 12 HHCs with microbiologically-confirmed TB who had drug-susceptibility testing for both isoniazid and rifampin did not have resistance to either drug, and were therefore discordant with their index participant’s resistance profile.

#### Factors associated with TB disease

After excluding the 8 HHCs with limited study evaluations and data collection because their TB diagnosis was made through the TB program, 704 HHCs age ≥15 years were studied to identify factors associated with TB disease by fitting univariable and multivariable models (see Table 3). Univariable models showed that prevalence of TB disease varied significantly by the HHC’s age, history of previous TB treatment, alcohol use in the past 12 months, smoking history, and the composite variable of incarceration, substance use, and/or alcohol use in the past 12 months.

**Table 3:** Logistic regression modeling for confirmed or probable TB disease in household contacts ≥15 years of age*.

		n/N (%) 38/704 (5%)	Univariable		Multivariable	
			Odds Ratio [95% CI]	P value	Odds Ratio [95% CI]	P value
HHC’s Age	15–24 years	7/189 (4%)	0.3 [0.1, 0.8]	**0.020**	0.5 [0.2, 1.1]	**0.011**
	25–49 years	14/341 (4%)	0.4 [0.2, 0.7]		0.3 [0.1, 0.6]	
	≥50 years	17/174 (10%)	1.0 (ref)		1.0 (ref)	
HHC’s Sex	Male	15/259 (6%)	1.1 [0.6, 2.2]	0.72		
	Female	23/445 (5%)	1.0 (ref)			
HHC’s HIV Status	HIV-positive	3/59 (5%)	0.9 [0.3, 2.7]	0.09	0.5 [0.1, 2.7]	0.07
	Unknown	3/130 (2%)	0.4 [0.1, 1.1]		0.3 [0.1, 1.0]	
	HIV-negative	32/515 (6%)	1.0 (ref)		1.0 (ref)	
HHC’s History of COPD/Asthma	Yes	3/29 (10%)	1.9 [0.6, 6.1]	0.41		
	No	34/661 (5%)	1.0 (ref)			
HHC’s History of Previous TB Treatment	Yes	21/77 (27%)	12.3 [6.3, 24.2]	**<0.001**	9.7 [4.5, 21.1]	**<0.001**
	No/Unknown	17/627 (3%)	1.0 (ref)		1.0 (ref)	
HHC Incarcerated Past 12 Mos.	Yes	1/20 (5%)	0.7 (0.1, 8.4)	0.76		
	No	37/683 (6%)	1.0 (ref)			
HHC Used Alcohol Almost Every Day Sometime in Past 12 Mos.	Yes	11/56 (20%)	5.5 [2.6, 11.7]	**0.008**	3.1 [1.3, 7.5]	**0.033**
	No	27/640 (4%)	1.0 (ref)		1.0 (ref)	
HHC Incarceration, Substance and/or Alcohol Use Composite in Past 12 Mos.^a^	Incarcerated	1/20 (5%)	1.2 [0.1, 10.0]	**0.023**		
	Never Incarcerated, Used Substances and/or Alcohol	14/82 (17%)	4.8 [2.3, 9.9]			
	Never Incarcerated, Did Not Use Substances or Alcohol	23/593 (4%)	1.0 (ref)			
HHC’s Smoking History	Current	20/151 (13%)	6.2 [3.1, 12.4]	**<0.001**	2.9 [1.3, 6.4]	**0.031**
	Previous	7/54 (13%)	6.3 [2.5, 16.0]		4.2 [1.5, 11.7]	
	Never	11/499 (2%)	1.0 (ref)		1.0 (ref)	
Index Participant’s Sputum Smear	AFB-positive	24/325 (7%)	2.1 [1.0, 4.4]	0.06		
	AFB-negative/Unknown	14/379 (4%)	1.0 (ref)			
Relationship of IP to HHC	Spouse	2/60 (3%)	0.6 [0.2, 2.1]	0.47		
	Mother	2/55 (4%)	0.5 [0.1, 2.6]			
	Father	1/37 (3%)	0.5 [0.1, 2.4]			
	Other	33/552 (6%)	1.0 (ref)			
Sleeping Proximity of IP to HHC	Same Room	15/240 (6%)	1.2 [0.6, 2.3]	0.69		
	Different Room	23/464 (5%)	1.0 (ref)			
Evenings/Week with IP, Past 6 Months	6–7	28/533 (5%)	1.1 [0.3, 3.4]	0.85		
	3–5	5/72 (7%)	1.5 [0.4, 6.4]			
	≤2	5/98 (5%)	1.0 (ref)			
Smokers in Household^b^	Any	24/273 (9%)	2.7 [1.3, 5.6]	**0.021**		
	None	14/422 (3%)	1.0 (ref)			
Primary Exterior Wall Material	Brick, Cinderblock, Stone with Mortar	25/544 (5%)	1.0 (ref)	0.22		
	Adobe, Rammed Earth, Sticks/Stones & Mud, Wood, Straw, Tin, Other	13/156 (8%)	1.8 [0.8, 4.2]			

IP, index participant; HHC, household contact; n, number of HHCs with positive IGRA result; N, number of HHCs with determinate IGRA result (positive or negative); %, simple percentage calculated as n/N; CI, confidence interval; ref, reference; mos., months.

* Models use data on HHC ≥15 years of age diagnosed with TB based on study evaluations; eight HHCs with active TB documented before study entry did not have data collection on incarceration, TB symptoms, or HIV status; they also did not have chest x-ray, specimen collection for IGRA, TB diagnostics, or HIV testing. Models were fit using an exchangeable working correlation, unless working correlation was negative (which occurred when data were sparse) in which case an independent working correlation was used. Univariable models include the one characteristic only. Each multivariable model includes characteristics with odds ratios shown. The p values are from score tests.

^a^ All self-reported. Incarceration is defined as having spent a night in jail, prison, youth detention center, alcohol use as having a period of drinking alcoholic beverages almost every day, and substance use as any use of substances (marijuana, cocaine, etc.) all in the past 12 months.

^b^ Smoking was defined as smoking tobacco products, ongoing or having quit within the past 6 months.

In multivariable modeling, compared to those who never smoked, HHCs who were current or previous smokers were more likely to have TB disease (aOR = 2.9 (95% CI: 1.3, 6.4) and aOR = 4.2 (95% CI: 1.5, 11.7), respectively). HHCs reporting a history of previous TB treatment were more likely to have prevalent TB disease, aOR = 9.7 (95% CI: 4.5, 21.1) compared to those without or with unknown history of TB treatment. HHCs who reported having used alcohol almost every day sometime in the past 12 months were more likely to have TB disease than those without such a period (aOR = 3.1 (95% CI: 1.3, 7.5)). Using the composite variable of incarceration, substance use, and/or alcohol use did not improve the model (not shown). We do not present models of predictors of TB disease for the subpopulation of HHCs who did not report a previous history of TB since more than half of those with TB disease had taken treatment for TB and few (n = 17) with TB disease would have contributed to the models.

## Discussion

We evaluated HHCs ≥15 years of age living in the household of an adult with rifampin-resistant pulmonary TB for prevalent Mtb infection and TB disease across 16 sites in eight countries from Africa (Botswana, Kenya, South Africa), Asia (India, Thailand), South America (Brazil, Peru), and the Caribbean (Haiti).

We found overlapping and distinct factors associated with prevalent Mtb infection and prevalent TB disease. Like others, we found that a previous history of TB treatment was associated with Mtb infection [7] and with TB disease [6,14,16–21]. A positive IGRA test could reflect *M*. *tb* sensitization in a person with longstanding latent *M*. *tb* infection, previous *M*. *tb* infection, or cured TB disease. Others have reported positive IGRA tests years after completion of anti-tuberculosis preventive therapy [22] and even decades after completing treatment of active TB disease [23]. We observed the highest prevalence of Mtb infection in HHCs 25–49 years of age, with a pattern similar to early TST surveys [24,25] and a 2002 report from The Gambia [11] of increasing prevalence with age and declines in older adults. We found that intensity of exposure to the index participant (sleeping proximity/evenings spent together) was associated with Mtb infection prevalence. Familial association and as intensity of exposure have been shown by others to be associated with Mtb infection [10,11,13,26]. Incarceration, which we found to be associated with Mtb infection, is likely a correlate of Mtb infection transmission from outside the household due to the high prevalence of TB in prisons and crowding. Incarceration is a well-established risk factor for TB [27]. Higher prevalence of Mtb infection was associated with exterior wall materials commonly used by lower socioeconomic status (SES) households. Lower SES has been associated with TB by others [28,29].

Smoking in the household was associated with higher prevalence of Mtb infection, but only individual-level current and previous smoking by the HHC was associated with higher prevalence of TB disease. Secondhand smoke has been shown by others to be associated with Mtb infection [30] and personal smoking with Mtb infection and TB disease [5,31]. Given the in vitro and murine data elucidating mechanisms by which smoking exposure increases susceptibility to *M*. *tuberculosis* [32], these results are not surprising and similar to results reported by others [31]. Higher prevalence of TB disease was also associated with self-report of a time when the HHC drank alcohol almost every day in the past 12 months. There is a consensus implicating heavy drinking and incidence of active TB [33], and our study adds to the evidence base, specifically implicating heavy alcohol use to higher prevalence of TB in the context of drug-resistant TB disease [34].

While many of these results are similar to other studies of Mtb infection and TB disease in presumed drug-susceptible TB, what is notable is that the study was large and multinational and used generally consistent definitions across countries to evaluate HHCs for factors associated with both Mtb infection and TB disease in HHCs of patients with drug-resistant TB.

Differing factors associated with prevalence of Mtb infection and TB disease are consistent with a two-step process with some distinct risk factors for Mtb infection and for developing disease described by Lienhardt [35]. However, some covariates may have been associated with Mtb infection but not TB disease because of being underpowered by the limited number of participants with TB disease.

Interestingly, when drug-susceptibility testing was done for HHCs with confirmed TB, the drug-resistance profiles showed the infecting organism was susceptible to isoniazid and rifampin in two-thirds. Whether this can be attributed to drug-susceptible minority strains being selectively transmitted from index participants or to the household contact investigation detecting previously undiagnosed TB disease resulting from exposure outside the household cannot be determined by the evaluations undertaken in this study. The discordance in drug-susceptibility profiles between index participants and HHCs we observed was higher than reported in a large meta-analysis that included household contacts from both low and high TB burden settings [36].

### Limitations

In non-TB endemic countries, it might be reasonable to assume that HHCs would have been exposed to *M*. *tb* through the index participant; however, this study was conducted in high TB burden countries so HHCs could have become infected through exposures outside the household.

IGRA, like TST, is an indirect marker of exposure to MTBC, which provides immunological evidence of sensitization to TB antigens, and has imperfect sensitivity [37]. As a result, we may have underestimated Mtb infection prevalence.

We enrolled index participants who started MDR-TB treatment in the past 6 months. The elapsed time since the index participant started treatment to enrollment of HHCs was a median of 9 weeks and varied from 0 to 33 weeks and will affect the prevalence of Mtb infection and TB disease. We may have missed some HHCs who eventually became infected with *M*. *tb* and/or progressed to disease as a result of exposure to the index participant.

## Conclusions

Prevalence of Mtb infection in HHCs ≥15 years of age exposed to patients with confirmed rifampin-resistant TB was high yet very few were receiving preventive treatment. This study may help programmatic staff identify HHCs at greatest risk of TB disease to rapidly link those with TB disease to care and inform individualized preventive treatment of Mtb infection consistent with current WHO guidance.

## Supporting information

S1 Table Institutional review boards and ethics committees overseeing A5300/I2003 PHOENIx feasibility study. (DOCX)

S2 Table Index participant and household characteristics by country of enrollment. Abbreviations: IP, index participant; n (%), number with attribute (percentage of participants); n/N (%), number with attribute/number with known or determinate for characteristic (percentage of known or determinate with attribute); Smear+, positive AFB smear result. † Includes the Index Participant and HHCs of all ages.
(DOCX)

S3 Table Characteristics of household contacts age ≥15 years by country of enrollment. Abbreviations: IP, index participant; HHC, household contact; n (%), number with attribute (percentage of participants); n/N (%), number with attribute/number with known or determinate for characteristic (percentage of known or determinate with attribute); ND, not done. ‡ IGRA data excludes 4 borderline results from both the numerator and denominator (2 from Peru and 2 from South Africa).
(DOCX)

S4 Table Elapsed years since the start of previous tuberculosis treatment if HHCs reported having been treated^a^. *Abbreviations and definitions: HHC, household contact; %, percentage; N, number; IQR, interquartile range (25^th^ and 75^th^ percentiles); range (minimum and maximum). For the range we do not present data more granular than <1 or >15 years to avoid potential disclosure of participant identity. Percentages do not add to 100% due to rounding. ^a^ The data collection instrument captured the year previous treatment for TB was started. Elapsed years was calculated by subtracting the year treatment started from the year of enrollment.
(DOCX)

S5 Table Isoniazid and rifampin drug susceptibility testing results in household contacts ≥15 years of age exposed to adults with pulmonary rifampin-resistant TB*. Abbreviations: TB, tuberculosis; INH, isoniazid; RIF, rifampin; DST, drug susceptibility testing. *Drug susceptibility testing was not done for 8 HHCs ≥15 years of age with active TB disease at study entry. Fourteen HHCs ≥15 years of age were determined to have TB based on study evaluations, and this table enumerates drug susceptibility to isoniazid and rifampin for these HHCs. MGIT, other phenotypic drug susceptibility testing, Xpert MTB/RIF, or HAIN GenoType MTBDR*plus* alone or in combination could have been used to determine the determine drug susceptibility.
(DOCX)

S1 File (DOCX)
