# Supplementary material for: Factors associated with prevalent Mycobacterium tuberculosis infection and disease among adolescents and adults exposed to rifampin-resistant tuberculosis in the household
Source: PLoS One. 2023 Mar 17;18(3):e0283290. doi: 10.1371/journal.pone.0283290 (PMC10022776; doi:10.1371/journal.pone.0283290)
Supplement: S1 Table — (DOCX) [file pone.0283290.s001.docx]

# **S1 Table. Institutional Review Boards and Ethics Committees Overseeing the A5300/I2003 PHOENIx Feasibility Study**

| **Clinical Research Site (CRS)** | **Institutional Review Board/Ethics Committee** |
| --- | --- |
| Gaborone CRS | Botswana Health Research & Development Committee (HRDC), Ministry of Health Government, Gaborone, Botswana; Harvard School of Public Health Office of Human Research Administration (OHRA), Harvard T. H Chan School of Public Health, Harvard Medical School, and Harvard School of Dental Medicine, Boston, MA USA |
| Instituto de Pesquisa Clinica Evandro Chagas CRS | Instituto Nacional de Infectilogia Evandro Chagas (INI), Rio de Janeiro, Brazil |
| GHESKIO Institute of Infectious Diseases and Reproductive Health (GHESKIO - IMIS) CRS | Comite National de Bioethique for IND trials, Comite des Droits Humains des Centres GHESKIO (CDH - GHESKIO); Haitian Global Health Alliance, Port-au-Prince, Haiti; Weill-Cornell Medical College (WCMC) IRB, Weill Cornell Medicine, New York, NY USA |
| Chennai Antiviral Research and Treatment (CART) CRS | Y.R. Gaitonde Centre for AIDS Research and Education (YRGCARE), Chennai, India |
| Byramjee Jeejeebhoy Government Medical College CRS | Johns Hopkins Medicine (JHM) IRB/US, Baltimore, MD USA; Byramjee Jeejeebhoy Medical College (BJMC) CTU, Pune, India; Health Ministry Screening Committee (HMSC), Ministry of Health and Family Welfare, Government of India |
| Barranco CRS | Impacta IRB, Lima, Peru |
| San Miguel CRS | Impacta IRB, Lima, Peru |
| University of the Witwatersrand Helen Joseph (WITS HJH) CRS | National Health Research Ethics Committee (NHREC), Department of Health, Republic of South Africa; Witswatersrand Health Research Ethics Committee (WITS HREC) Johannesburg, South Africa; Provincial - Project and Programme Review Committee (Provincial - PPRC), South Africa |
| Durban International CRS | South Africa Pharma Ethics; Independent Research Ethics Committee in South Africa, KwaZulu-Natal Department of Health (KZN DOH), KwaZulu-Natal, South Africa |
| Soweto ACTG CRS | Witswatersrand Health Research Ethics Committee (WITS HREC), Johannesburg, South Africa |
| TASK Applied Science CRS | Pharma Ethics Independent Research Ethics Committee (IREC), Independent Research Ethics Committee in South Africa |
| Desmond Tutu TB Centre - Stellenbosch University (DTTC-SU) CRS | Human Research Ethics Committee (HREC), Stellenbosch University, South Africa |
| University of Cape Town Lung Institute (UCTLI) CRS | University of Cape Town, Faculty of Health Sciences, Human Research Ethics Committee (UCT-FHS-HREC), Cape Town, South Africa; Department of Health Clinical Trials - (DOH-CT), South Africa |
| South African Tuberculosis Vaccine Initiative (SATVI) CRS | Scientific Protocol Review Committee, University of Cape Town, Faculty of Health Sciences, Human Research Ethics Committee (UCT-FHS-HREC), Cape Town, South Africa; Western Cape Department of Health (DOH), South Africa |
| Chiangrai Prachanukroh Hospital NICHD CRS | Ethical Review Committee (ERC) for Research in Human Subjects, Ministry of Public Health, Thailand |
| Kenya Medical Research Institute/Center for Disease Control (KEMRI/CDC) CRS | Independent Scientific and Ethics Review Unit (SERU), Center for Global Health Research Center Scientific Committee (CSC), Nairobi, Kenya |
