# Supplementary material for: Factors associated with prevalent Mycobacterium tuberculosis infection and disease among adolescents and adults exposed to rifampin-resistant tuberculosis in the household
Source: PLoS One. 2023 Mar 17;18(3):e0283290. doi: 10.1371/journal.pone.0283290 (PMC10022776; doi:10.1371/journal.pone.0283290)
Supplement: S2 Table — Abbreviations: IP, index participant; n (%), number with attribute (percentage of participants); n/N (%), number with attribute/number with known or determinate for characteristic (percentage of known or determinate with attribute); Smear+, positive AFB smear result. † Includes the Index Participant and HHCs of all ages. (DOCX) [file pone.0283290.s002.docx]

# **S2 Table. Index Participant and Household Characteristics by Country of Enrollment**

| **Country** | **Number of IP** | **Age (years): median (IQR)** | **Female: n (%)** | **Weeks IP on TB Treatment at Enrollment: median (IQR)** | **HIV Positive:   n/N (%)** | **Sputum Smear+ at Diagnosis: n/N (%)** | **Cavitations on Chest X-ray n/N (%)** | **Household Size†: median (IQR)** |
| --- | --- | --- | --- | --- | --- | --- | --- | --- |
| Botswana | 10 | 38 (33, 52) | 6 (60%) | 4 (<1, 25) | 6/10 (60%) | 7/8 (88%) | 4/9 (44%) | 4.5 (4, 6) |
| Brazil | 10 | 45.5 (36, 48) | 2 (20%) | 15 (11, 18) | 0/8 (0%) | 7/8 (88%) | 2/4 (50%) | 4 (4, 6) |
| Haiti | 14 | 40 (27, 52) | 7 (50%) | 8 (2, 20) | 5/13 (38%) | 7/10 (70%) | 1/2 (50%) | 4 (2, 5) |
| India | 58 | 32 (25, 42) | 19 (33%) | 10 (5, 16) | 4/36 (11%) | 2/2 (100%) | 34/44 (77%) | 4 (3, 6) |
| Kenya | 7 | 36 (29, 41) | 1 (14%) | 12 (4, 22) | 6/7 (86%) | 3/4 (75%) | 5/7 (71%) | 4 (3, 4) |
| Peru | 51 | 24 (20, 36) | 17 (33%) | 10 (5, 16) | 1/49 (2%) | 45/50 (90%) | 6/25 (24%) | 5 (4, 7) |
| South Africa | 120 | 38 (29, 44) | 63 (53%) | 7 (2, 16) | 77/119 (65%) | 52/102 (51%) | 46/81 (57%) | 5 (3, 6.5) |
| Thailand | 9 | 48 (40, 60) | 1 (11%) | 14 (12, 17) | 2/9 (22%) | 8/8 (100%) | 0/5 (0%) | 5 (4, 8) |
| **Overall** | 279 | 36 (26, 45) | 116 (42%) | 9 (3, 17) | 101/251 (40%) | 131/192 (68%) | 98/177 (55%) | 5 (3, 6) |

Abbreviations: IP, index participant; n (%), number with attribute (percentage of participants); n/N (%), number with attribute/number with known or determinate for characteristic (percentage of known or determinate with attribute); Smear+, positive AFB smear result.

† Includes the Index Participant and HHCs of all ages.
