# Supplementary material for: Factors associated with prevalent Mycobacterium tuberculosis infection and disease among adolescents and adults exposed to rifampin-resistant tuberculosis in the household
Source: PLoS One. 2023 Mar 17;18(3):e0283290. doi: 10.1371/journal.pone.0283290 (PMC10022776; doi:10.1371/journal.pone.0283290)
Supplement: S3 Table — Abbreviations: IP, index participant; HHC, household contact; n (%), number with attribute (percentage of participants); n/N (%), number with attribute/number with known or determinate for characteristic (percentage of known or determinate with attribute); ND, not done. ‡ IGRA data excludes 4 borderline results from both the numerator and denominator (2 from Peru and 2 from South Africa). (DOCX) [file pone.0283290.s003.docx]

**S3 Table. Characteristics of Household Contacts Age ≥15 Years by Country of Enrollment**

| **Country** | **Number of HHCs Age ≥15 years** | **Female: n (%)** | **Previously Treated for TB: n (%)** | **Weeks IP on TB Treatment at HHC Enrollment: median (IQR)** | **HIV Positive: n/N (%)** | **TST Positive: n/N (%)** | **IGRA Positive^‡^: n/N (%)** |
| --- | --- | --- | --- | --- | --- | --- | --- |
| Botswana | 20 | 2 (10%) | 2 (10%) | 4 (2, 18) | 1/12 (8%) | 2/20 (10%) | 12/19 (63%) |
| Brazil | 17 | 5 (29%) | 2 (12%) | 15 (12, 18) | 0/12 (0%) | 7/9 (78%) | 12/17 (71%) |
| Haiti | 37 | 14 (38%) | 2 (5%) | 6 (2, 14) | 1/37 (3%) | 14/33 (42%) | 26/37 (70%) |
| India | 170 | 75 (44%) | 6 (4%) | 8 (5, 16) | 6/157 (4%) | 86/141 (61%) | 100/168 (60%) |
| Kenya | 12 | 0 (0%) | 1 (8%) | 12 (5, 21) | 4/12 (33%) | 4/8 (50%) | 6/8 (75%) |
| Peru | 130 | 48 (37%) | 13 (10%) | 12 (6, 19) | 0/63 (0%) | ND | 81/126 (64%) |
| South Africa | 301 | 109 (36%) | 53 (18%) | 9 (4, 18) | 44/256 (17%) | 175/258 (68%) | 221/286 (77%) |
| Thailand | 25 | 10 (40%) | 1 (4%) | 12 (2, 16) | 3/25 (12%) | 4/25 (16%) | 13/25 (52%) |
| **Overall** | 712 | 263 (37%) | 80 (11%) | 9 (4, 18) | 59/574 (10%) | 292/494 (59%) | 471/686 (69%) |

Abbreviations: IP, index participant; HHC, household contact; n (%), number with attribute (percentage of participants); n/N (%), number with attribute/number with known or determinate for characteristic (percentage of known or determinate with attribute); ND, not done.

‡ IGRA excludes 4 borderline results from both the numerator and denominator (2 from Peru and 2 from South Africa).
