# Supplementary material for: Factors associated with prevalent Mycobacterium tuberculosis infection and disease among adolescents and adults exposed to rifampin-resistant tuberculosis in the household
Source: PLoS One. 2023 Mar 17;18(3):e0283290. doi: 10.1371/journal.pone.0283290 (PMC10022776; doi:10.1371/journal.pone.0283290)
Supplement: S4 Table — *Abbreviations and definitions: HHC, household contact; %, percentage; N, number; IQR, interquartile range (25th and 75th percentiles); range (minimum and maximum). For the range we do not present data more granular than <1 or >15 years to avoid potential disclosure of participant identity. Percentages do not add to 100% due to rounding. a The data collection instrument captured the year previous treatment for TB was started. Elapsed years was calculated by subtracting the year treatment started from the year of enrollment. (DOCX) [file pone.0283290.s004.docx]

**S4 Table. Elapsed Years since the Start of Previous Tuberculosis Treatment if HHCs Reported Having Been Treated^*^**

| **Elapsed years since start of previous treatment: median (range), IQR, N (%)** | **N=80** |
| --- | --- |
| median (range) | 7 (<1, >15) |
| IQR | (2, 16) |
| <1 | 2 (3%) |
| 1-2 | 18 (23%) |
| 3-5 | 16 (21%) |
| 6-15 | 21 (27%) |
| >15 | 21 (27%) |
| Missing | 2 |

Abbreviations and definitions: HHC, household contact; %, percentage; N, number; IQR, interquartile range (25^th^ and 75^th^ percentiles); range (minimum and maximum). For the range we do not present data more granular than <1 or >15 years to avoid potential disclosure of participant identity. Percentages do not add to 100% due to rounding.

* The data collection instrument captured the year previous treatment for TB was started. Elapsed years was calculated by subtracting the year treatment started from the year of enrollment.
