# Supplementary material for: Factors associated with prevalent Mycobacterium tuberculosis infection and disease among adolescents and adults exposed to rifampin-resistant tuberculosis in the household
Source: PLoS One. 2023 Mar 17;18(3):e0283290. doi: 10.1371/journal.pone.0283290 (PMC10022776; doi:10.1371/journal.pone.0283290)
Supplement: S5 Table — Abbreviations: TB, tuberculosis; INH, isoniazid; RIF, rifampin; DST, drug susceptibility testing. *Drug susceptibility testing was not done for 8 HHCs ≥15 years of age with active TB disease at study entry. Fourteen HHCs ≥15 years of age were determined to have TB based on study evaluations, and this table enumerates drug susceptibility to isoniazid and rifampin for these HHCs. MGIT, other phenotypic drug susceptibility testing, Xpert MTB/RIF, or HAIN GenoType MTBDRplus alone or in combination could have been used to determine the determine drug susceptibility. (DOCX) [file pone.0283290.s005.docx]

**S5 Table. Isoniazid and Rifampin Drug Susceptibility Testing Results in Household Contacts ≥15 Years of Age Exposed to Adults with Pulmonary Rifampin-Resistant TB***

| **INH and RIF Drug Susceptibility** | **Frequency**  **(N=14)** |
| --- | --- |
| INH Resistant, RIF Resistant | 4 |
| INH Susceptible, RIF Susceptible | 8 |
| INH Unknown, RIF Susceptible | 1 |
| Unknown (no DST results) | 1 |

Abbreviations: TB, tuberculosis; INH, isoniazid; RIF, rifampin; DST, drug susceptibility testing.

*Drug susceptibility testing was not done for 8 HHCs ≥15 years of age with active TB disease at study entry. Fourteen HHCs ≥15 years of age were determined to have TB based on study evaluations, and this table enumerates drug susceptibility to isoniazid and rifampin for these HHCs. MGIT, other phenotypic drug susceptibility testing, Xpert MTB/RIF, or HAIN GenoType MTBDR*plus* alone or in combination could have been used to determine the determine drug susceptibility.
